# Sarcopenia is associated with leukopenia in urothelial carcinoma patients who receive tislelizumab combined with gemcitabine and cisplatin therapy

**DOI:** 10.1007/s10147-023-02448-1

**Published:** 2024-03-22

**Authors:** Zhimin Gao, Yubin Pang, Xu Qin, Gang Li, Zewei Wang, Lei Zhang, Junqi Wang, Nienie Qi, Hailong Li

**Affiliations:** 1 Department of Urology, The Affiliated Hospital of Xuzhou Medical University, Xuzhou, 221000 People’s Republic of China; 2 Graduate School of Xuzhou Medical University, Xuzhou, 221000 People’s Republic of China; 3 Suining People’s Hospital, Xuzhou, 221000 People’s Republic of China

**Keywords:** Urothelial carcinoma, Body composition, Sarcopenia, Tislelizumab, Toxicity, Tumor response

## Abstract

**Background:**

In the era of combination therapy, there has been limited research on body composition. Specific body composition, such as sarcopenia, possesses the potential to serve as a predictive biomarker for toxic effects and clinical response in patients with urothelial carcinoma (UC) undergoing tislelizumab combined with gemcitabine and cisplatin (T + GC).

**Materials and Methods:**

A total of 112 UC patients who received T + GC were selected at the Affiliated Hospital of Xuzhou Medical University from April 2020 to January 2023. Baseline patient characteristics and detailed hematological parameters were collected using the electronic medical system and laboratory examinations. The computed tomography images of patients were analyzed to calculate psoas muscle mass index (PMI). We evaluated the association between sarcopenia (PMI < 4.5 cm^2^/m^2^ in men; PMI < 3.3 cm^2^/m^2^ in women) and both hematological toxicity and tumor response.

**Results:**

Overall, of the 112 patients (65.2% male, median age 56 years), 43 (38.4%) were defined as sarcopenia. Patients with sarcopenia were notably older (*p* = 0.037), more likely to have hypertension (*p* = 0.009), and had poorer ECOG-PS (*p* = 0.027). Patients with sarcopenia were more likely to develop leukopenia (OR 2.969, 95% CI 1.028–8.575, *p* = 0.044) after receiving at least two cycles of T + GC. However, these significant differences were not observed in thrombocytopenia and anemia. There were no significant differences in the tumor response and grade 3–4 hematological toxicity between patients with sarcopenia and those without sarcopenia.

**Conclusions:**

Patients with sarcopenia were more likely to develop leukopenia after receiving T + GC. There were no notable alterations observed in relation to anemia or thrombocytopenia. No significant difference was found between the sarcopenia group and non-sarcopenia group in terms of tumor response and grade 3–4 hematological toxicity.

## Introduction

With the advent of immune checkpoint inhibitors (ICIs), patients with locally advanced and metastatic urothelial carcinoma (UC) have a broader range of treatment options. Therefore, an increasing number of studies focus on the therapeutic effects of immunotherapy combined with chemotherapy. It is worth mentioning that the adverse effects (AEs) of combination therapy cannot be ignored. This underscores the urgent need for research into predictive and easily measurable biomarkers to identify suitable patients with UC for this treatment approach.

Sarcopenia is the most frequently assessed parameter for evaluating complex body composition and has significant clinical value. Sarcopenia refers to the loss of skeletal muscle mass and function, which is believed to be linked to perioperative complications [1, 2] and a negative prognosis in UC [3–7]. However, whether sarcopenia has an effect on treatment-related adverse events (TRAEs), particularly hematological toxicity, in UC patients receiving immunochemotherapy remains a subject of debate. Hence, we conducted a retrospective study to determine whether there is an association between pre-existing sarcopenia in UC patients before receiving immunochemotherapy and the incidence of treatment-related hematological toxicity as well as tumor response rates.

## Materials and methods

### Patients selection

From April 2020 to January 2023, 112 patients with UC who received first-line tislelizumab plus gemcitabine and cisplatin (T + GC) therapy at our institution were retrospectively evaluated. Eligible patients had histologically confirmed UC of the renal pelvis, ureter, or bladder and received at least two cycles of T + GC. Adequate laboratory examinations, including baseline hematological parameters and post-treatment complete blood count (CBC), were required.

**Fig. 1:**
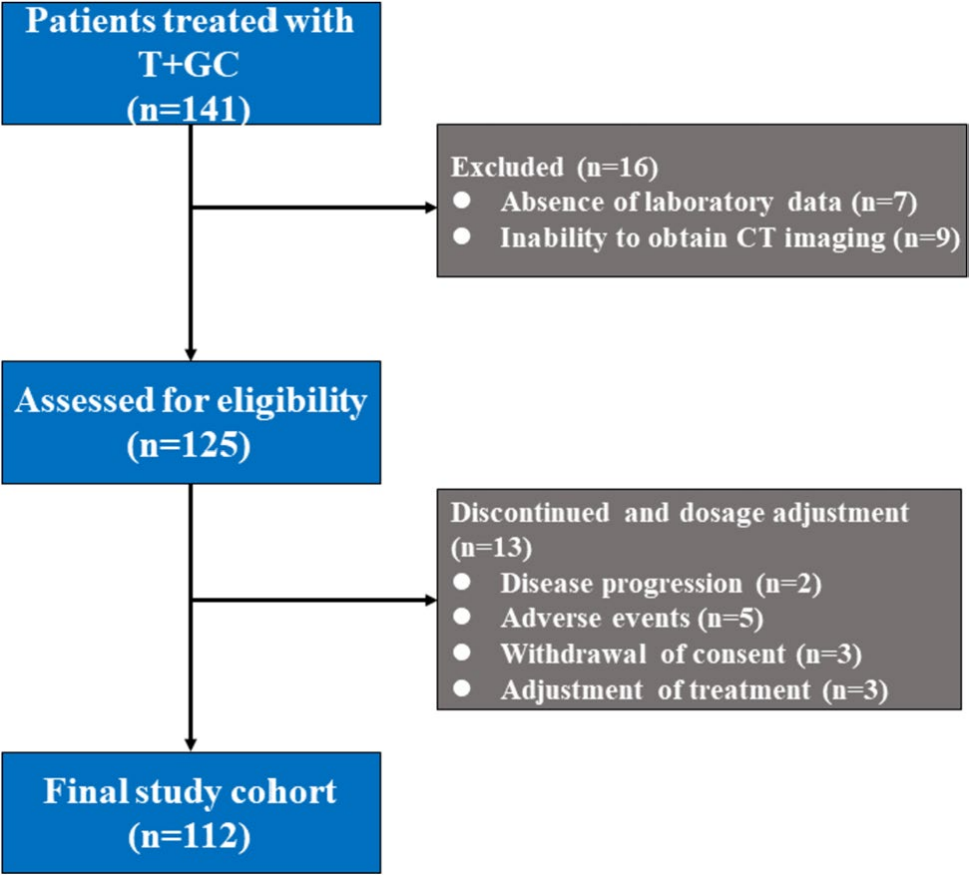
Flowchart showing patient selection

Exclusion criteria included the inability to obtain computed tomography (CT) imaging. Patients who had previously received treatment with ICIs or cytotoxic chemotherapy should be excluded. Patients who had received prophylactic corticosteroids or other medications to promote hematopoiesis within 1 month prior to T + GC administration should also be excluded. A total of 112 patients met criteria (Fig. 1).

Ethical approval was granted by the Ethics Committee of the Affiliated Hospital of Xuzhou Medical University (approval number: XYFY2022-KL340). All patients provided written informed consent.

### Definition of sarcopenia

CT has been established as a reliable method for accurately measuring of body composition parameters [8], such as skeletal muscle and subcutaneous adipose tissue. We utilized the psoas muscle mass index (PMI) at the level of the third lumbar vertebra (L3) in CT images as an alternative indicator for evaluating skeletal muscle mass. The cross-sectional area (CSA) of the bilateral psoas muscles was calculated, and the mean value for two consecutive images was computed for each patient. All images were analyzed by two independent researchers using ImageJ software (Version 1.49, National Institutes of Health, USA) based on the aforementioned criteria. PMI (cm^2^/m^2^) was calculated using this formula: the mean CSA of psoas muscle (cm^2^) from two consecutive CT images / the square of the body height (m^2^).

A study based on a large sample of the Asian population demonstrated that the PMI cut-off value for sarcopenia in male patients was 6.36 cm^2^/m^2^, while in female patients it was 3.92 cm^2^/m^2^ [9]. In this study, sarcopenia was defined as less than 2 standard deviations (SDs) below the mean PMI of healthy Asian adults. However, our research focused on patients diagnosed with UC who were predominantly of older age, and almost all of them were classified as sarcopenia according to the above definition. Therefore, we adopted the method of selecting cut-off values for sarcopenia from previous studies [10] and utilized a cut-off value of > 1 SD below our mean value (< 4.5 cm^2^/m^2^ for male patients; < 3.3 cm^2^/m^2^ for female patients) to identify individuals with sarcopenia.

### Data collection and evaluations

The clinicopathological data were collected from the medical records, including age, sex, body mass index (BMI), smoking history, hypertension, diabetes, hydronephrosis, Eastern Cooperative Oncology Group performance score (ECOG PS), primary tumor site (renal pelvis/ureter/bladder) and renal function. The CT scan within three months before the start of T + GC and throughout treatment should be collected.

The laboratory parameters include white blood cell (WBC) count, hemoglobin level, and platelet count. Patients with renal insufficiency need to meet both criteria of blood creatinine > 133 μmol/l and eGFR > 60 ml/min. The incidence and severity of TRAEs were assessed by Common Terminology Criteria for Adverse Events (CTCAE) Version 4.03 of the National Cancer Institute.

### Drug administration and the evaluation of responses

The patients received a 21-day cycle of treatment. Patients were administered intravenously gemcitabine (1000 mg per square meter of body-surface area) on days 1 and 8, in combination with cisplatin (70 mg per square meter of body-surface area) and tislelizumab (200 mg) on day 1 as first-line therapy. According to the pathological stages, the treatment cycle of T + GC varied. For patients with locally advanced UC, four cycles of postoperative adjuvant therapy were administered. For patients with metastatic UC, six cycles of systemic therapy were recommended. The combination therapy was terminated permanently until the completion of treatment schedule, disease progression, intolerable toxicity, or withdrawal of consent.

Response evaluation of the target lesions was performed via CT scan using the Response Evaluation Criteria in Solid Tumors (RECIST) version 1.1 [11]. The first response evaluation was performed after two cycles of T + GC, followed by a reassessment every two treatment cycles.

### Statistical analyses

All the data were transformed into categorical variables, and descriptive statistics, such as percentages, means, and medians were used to report baseline characteristics of patients. After testing for normal distribution, the Chi-square test and Fisher's exact test were used to investigate the differences between the sarcopenia group and the non-sarcopenia group. For non-normally distributed variables, the Wilcoxon–Mann–Whitney *U* test was performed. Univariate and multivariate logistic regression were conducted to examine potential risk factors. The results were reported as odds ratios (OR) with 95% confidence intervals (CI). All analyses were performed using SPSS 26.0 (IBM Corp., NY, USA). All statistical analyses were two-sided, and statistical significance was set at *p* < 0.05.

## Results

### Descriptive data

From April 2020 to January 2023, a total of 112 patients with UC received at least two cycles of T + GC. The patient characteristics are shown in Table 1. The median age was 56 (range 34–82), with 39 (34.8%) females and 73 (65.2%) males. Among the study population, 38.4% of patients were classified as sarcopenia. The age of patients with sarcopenia exhibited a significant increase (*p* = 0.037), and the median age of patients in the sarcopenia group and non-sarcopenia group was 63 years and 54 years, respectively. Additionally, patients with sarcopenia were more likely to have hypertension (*p* = 0.009), and had poorer ECOG PS (*p* = 0.027). Patients with sarcopenia were not associated with a significant reduction in BMI.

**Table 1:** Patient demographics and baseline disease characteristics

Characteristic	Total *N* = 112	Non-Sarcopenia * N* = 69	Sarcopenia *N* = 43	*p* Value
Median age[range], years	56 [34–82]	54 [42–77]	63 [34–82]	
Age group, n (%)				0.037
≤ 70	78 (69.6)	53 (76.8)	25 (74.4)	
> 70	34 (30.4)	16 (23.2)	18 (25.6)	
Sex, n (%)				0.675
Female	39 (34.8)	23 (33.3)	16 (37.2)	
Male	73 (65.2)	46 (66.7)	27 (62.8)	
BMI, n (%)				0.621
< 18.5	5 (4.5)	4 (5.8)	1 (2.3)	
18.5–24.9	62 (55.4)	36 (52.2)	26 (60.5)	
25–29.9	36 (32.1)	22 (31.9)	14 (32.6)	
> 30	9 (8.0)	7 (10.1)	2 (4.7)	
Smoking history, n (%)				0.794
No	40 (35.7)	24 (34.8)	16 (37.2)	
Yes	72 (64.3)	45 (65.2)	27 (62.8)	
Hypertension				0.009
No	83 (74.1)	57 (82.6)	26 (60.5)	
Yes	29 (25.9)	12 (17.4)	17 (39.5)	
Diabetes				0.116
No	97 (86.6)	57 (82.6)	40 (93.0)	
Yes	15 (13.4)	12 (17.4)	3 (7.0)	
Hydronephrosis, n (%)				0.197
No	34 (30.4)	24 (34.8)	10 (23.3)	
Yes	78 (69.6)	45 (65.2)	33 (76.7)	
ECOG status, n (%)				0.027
0	81 (72.3)	55 (79.7)	26 (60.5)	
1	31 (27.7)	14 (20.3)	17 (39.5)	
Primary tumor site, n (%)				0.794
UTUC	66 (58.9)	40 (58.0)	26 (60.5)	
BC	46 (41.1)	29 (42.0)	17 (39.5)	
Surgical removal of primary site, n (%)				0.802
No	7 (6.2)	4 (5.8)	3 (7.0)	
Yes	105 (93.8)	65 (94.2)	40 (93.0)	
T stage				0.792
T2	41 (36.6)	25 (36.2)	16 (37.2)	
T3	43 (38.4)	28 (40.6)	15 (34.9)	
T4	28 (25.0)	16 (23.2)	12 (27.9)	
N stage				0.448
Nx	6 (5.4)	3 (4.3)	3 (7.0)	
N0	62 (55.4)	36 (52.2)	26 (60.4)	
*N* ≥ 1	44 (39.2)	30 (43.5)	14 (32.6)	
M stage				0.169
Mx	4 (3.6)	3 (4.3)	1 (2.3)	
M0	91 (81.2)	59 (85.5)	32 (74.4)	
M1	17 (15.2)	7 (10.2)	10 (23.3)	
Renal dysfunction, n (%)				0.447
No	90 (80.4)	57 (82.6)	33 (76.7)	
Yes	22 (19.6)	12 (17.4)	10 (23.3)	

BMI, body mass index; ECOG, Eastern Cooperative Oncology Group; UTUC, upper tract urothelial carcinoma; BC, bladder cancer

### Sarcopenia and hematological toxicity

The majority of patients (*n* = 79; 70.5%) developed anemia during treatment. Among them, 47 (59.5%) patients developed anemia immediately after the first cycle of T + GC. After receiving one cycle of T + GC, patients with sarcopenia (*n* = 24; 55.8%) were more likely to develop anemia than those without sarcopenia (*n* = 23; 33.3%). Among patients receiving adjuvant treatment after radical surgery, 30 (75.0%) patients in the sarcopenia group experienced postoperative anemia, whereas 44 (67.7%) patients in the non-sarcopenia group developed anemia. None of the patients underwent dose adjustment due to anemia. One patient permanently discontinued treatment due to grade 4 anemia and received blood transfusion therapy. Patients with sarcopenia were more likely to develop grade 3–4 anemia (25.6 vs. 11.6%, *p* = 0.055), but this did not reach statistical significance (Table 2). Multivariate logistic regression analysis showed that the occurrence of anemia was associated with the age (OR 7.921, 95% CI 1.078–58.185, *p* = 0.042) of UC patients (Table 3).

**Table 2:** High-grade hematological toxicities reported for patients of the sarcopenia and non-sarcopenia arms

Hematological AEs	Non-sarcopenia (*n* = 69)					Sarcopenia (*n* = 43)				
	Grade 3			Grade 4		Grade 3			Grade 4	
	No. of patients	%		No. of patients	%	No. of patients	%		No. of patients	%
Anemia	8	11.6		0	0	10	23.3		1	2.3
Leukopenia	18	26.1		3	4.3	14	32.6		3	7.0
Thrombocytopenia	6	8.7		1	1.4	6	14.0		2	4.7

**Table 3:** Logistic regression model of factors predicting anemia

Variables	Univariate analysis		Multivariate analysis	
	OR (95% CI)	*p*	OR (95% CI)	*p*
Age				
≤ 70	1		1	
> 70	3.414 (1.024–11.380)	0.046	7.921 (1.078–58.185)	0.042
BMI				
< 25	1			
≥ 25	0.909 (0.343–2.411)	0.848		
Smoking history				
Never	1			
Ever	0.933 (0.337–2.585)	0.894		
Hydronephrosis				
No	1			
Yes	1.437 (0.521–3.961)	0.483		
ECOG status				
0	1			
1	1.611 (0.513–5.056)	0.414		
Primary tumor site				
UTUC	1			
BC	0.623 (0.233–1.670)	0.347		
Surgical removal of primary site				
No	1			
Yes	1.651 (0.257–10.600)	0.597		
Renal dysfunction				
No	1			
Yes	1.481 (0.368–5.969)	0.580		
Body composition				
Non-sarcopenia	1			
Sarcopenia	1.250 (0.454–3.444)	0.666		
Treatment cycle				
1	1			
≥ 2	1.250 (0.454–3.444)	0.666		

Of the 112 UC patients who received T + GC, 44 (39.3%) developed thrombocytopenia. Compared to patients who did not undergo surgical treatment (*n* = 3; 2.7%), patients who underwent radical surgery were more likely to develop thrombocytopenia (*n* = 21; 18.8%) after multiple cycles of T + GC. Compared with the non-sarcopenia group, patients with sarcopenia were more likely to develop grade 3–4 thrombocytopenia (18.7 vs. 10.1%, *p* = 0.201). However, this difference did not reach the threshold for statistical significance (Table 2). Two patients permanently discontinued T + GC therapy due to severe thrombocytopenia, and one patient received a platelet transfusion. Results from multivariate logistic regression analysis showed that smoking history (OR 3.294, 95% CI 1.022–10.615, *p* = 0.046) was associated with thrombocytopenia in patients (Table 4).

**Table 4:** Logistic regression model of factors predicting thrombocytopenia

Variables	Univariate analysis		Multivariate analysis	
	OR (95% CI)	*p*	OR (95% CI)	*p*
Age				
≤ 70	1			
> 70	1.066 (0.412–2.759)	0.896		
BMI				
< 25	1			
≥ 25	0.484 (0.190–1.230)	0.127		
Smoking history				
Never	1		1	
Ever	2.130 (0.791–5.736)	0.135	3.294 (1.022–10.615)	0.046
Hydronephrosis				
No	1			
Yes	1.385 (0.521–3.681)	0.514		
ECOG status				
0	1			
1	0.820 (0.296–2.271)	0.702		
Primary tumor site				
UTUC	1			
BC	0.590 (0.229–1.521)	0.274		
Surgical removal of primary site				
No	1			
Yes	2.791 (0.297–26.223)	0.369		
Renal dysfunction				
No	1			
Yes	1.371 (0.413–4.551)	0.606		
Body composition				
Non-sarcopenia	1			
Sarcopenia	0.643 (0.249–1.661)	0.362		
Treatment cycle				
1	1			
≥ 2	0.610 (0.245–1.521)	0.289		

Of the 112 patients with UC, half of the patients (*n* = 56; 50%) experienced leukopenia during all cycles of T + GC. Among patients with leukopenia, there were 9 (16.1%) patients in the non-sarcopenia group and 13 (23.2%) patients in the sarcopenia group who had pre-existing renal insufficiency prior to the start of T + GC. 28 (25.0%) patients treated with short-acting granulocyte colony stimulating factor, the WBC count rebounded enough to tolerate the next cycle of T + GC, and three patients with sarcopenia permanently discontinued therapy due to severe leukopenia. A statistically insignificant higher incidence of grade 3 or 4 leukopenia occurred in the sarcopenia group (39.6 vs. 30.4%, *p* = 0.324) (Table 2). The results from multivariate logistic regression analysis showed that patients with sarcopenia were associated with leukopenia after receiving T + GC (OR 2.969, 95% CI 1.028–8.575, *p* = 0.044) (Table 5). For UC patients received T + GC, patients with sarcopenia were more likely to develop leukopenia.

**Table 5:** Logistic regression model of factors predicting leukopenia

Variables	Univariate analysis		Multivariate analysis	
	OR (95% CI)	*p*	OR (95% CI)	*p*
Age				
≤ 70	1			
> 70	1.406 (0.551–3.590)	0.476		
BMI				
< 25	1			
≥ 25	1.229 (0.504–2.998)	0.650		
Smoking history				
Never	1			
Ever	1.565 (0.616–3.977)	0.346		
Hydronephrosis				
No	1			
Yes	1.260 (0.490–3.237)	0.631		
ECOG status				
0	1			
1	1.000 (0.373–2.681)	1.000		
Primary tumor site				
UTUC	1			
BC	1.113 (0.449–2.758)	0.817		
Surgical removal of primary site				
No	1			
Yes	1.542 (0.243–9.776)	0.646		
Renal dysfunction				
No	1			
Yes	2.422 (0.882–6.650)	0.086		
Body composition				
Non-sarcopenia	1		1	
Sarcopenia	3.053 (1.175–7.928)	0.022	2.969 (1.028–8.575)	0.044
Treatment cycle				
1	1			
≥ 2	0.658 (0.267–1.619)	0.362		

### Sarcopenia and clinical response

During the treatment period, a total of 45 patients with UC had at least one lesion that could be evaluated and measured (Table 6). Of the 45 patients treated with T + GC, 11(24.4%) exhibited a partial response, 19(42.2%) experienced stable disease, and only one patient (2.2%) achieved a complete response. The objective response rate (ORR) and disease control rate (DCR) in the entire cohort were 26.7% and 68.9%, respectively. Patients with sarcopenia had a higher ORR than those without sarcopenia, although the difference did not reach statistical significance (31.3 vs. 24.1%, *p* = 0.606). The DCR in the sarcopenia group and non-sarcopenia group was 68.8% and 68.9%, respectively.

**Table 6:** Comparison of disease response in patients with and without sarcopenia

Response category	Non-sarcopenia (*n* = 29)	Sarcopenia (*n* = 16)	*p* value
Complete response, n (%)	1 (3.4)	0 (0)	
Partial response, n (%)	6 (20.7)	5 (31.3)	
Stable disease, n (%)	13 (44.8)	6 (37.5)	
Progressive disease, n (%)	9 (31.0)	5 (31.3)	
ORR (%, CR + PR)	24.1	31.3	0.606
DCR (%, CR + PR + SD)	68.9	68.8	0.988

CR, complete response; PR, partial response; SD, stable disease; PD, progressive disease; ORR, objective response rate; DCR, disease control rate

## Discussion

Since the 1980s, cisplatin-based chemotherapy has been the first-line standard regimen for metastatic UC [12]. The European Association of Urology (EAU) guidelines recommended neoadjuvant chemotherapy (NAC) for T2-T4aN0M0 bladder cancer. If NAC is not performed, adjuvant chemotherapy (AC) is required [13]. For patients with advanced upper tract urothelial carcinoma (UTUC), postoperative AC is recommended [14]. However, despite effective treatment, the prognosis of muscle-invasive UC remains poor with 5-year survival rates less than 60% [15, 16]. The standard first-line chemotherapy in the treatment of muscle-invasive UC provides only a modest survival benefit.

With the emergence of ICIs, patients with locally advanced and metastatic UC have more therapeutic options. The Phase III trial, IMvigor130 [17], demonstrated that immunotherapy combined with chemotherapy, compared to first-line platinum-based chemotherapy, significantly improved progression-free survival (PFS) for patients with UC (*p* = 0.007). The CheckMate-901 trial revealed that for patients with unresectable or metastatic UC, the combination of nivolumab with cisplatin-based chemotherapy followed by nivolumab monotherapy demonstrated statistically significant benefits in OS and PFS compared to first-line cisplatin-based chemotherapy. In April 2020, tislelizumab was approved for the treatment of advanced UC with high expression of programmed death-ligand 1 (PD-L1), marking the beginning of a new era in immunotherapy for UC in China. Current researches indicate that in the neoadjuvant [18] or adjuvant treatment [19] of locally advanced or metastatic bladder cancer, the combination of T + GC has yielded satisfactory clinical outcomes. Therefore, there is an urgent need to find easy-to-measure and predictive parameters that can predict the risk of toxic reactions and tumor response in advance.

Sarcopenia is a condition characterized by the systemic deterioration of muscle function and mass. It is observed in approximately 15–50% of patients aged 65 and older, and it is particularly prevalent in patients with advanced tumors [20]. Compared to the skeletal muscle index (SMI), the PMI is easier to obtain with sonography [21], making it commonly used as an alternative indicator for assessing sarcopenia [22]. Many studies have demonstrated that sarcopenia predominantly has a negative impact on the prognosis of UC patients who undergo radical resection or chemotherapy [23–25]. Furthermore, sarcopenia is thought to be associated with an increased incidence of postoperative complications [26, 27].

There is currently an ongoing debate regarding whether pre-existing sarcopenia prior to immunotherapy increases the risk of AEs. In patients with metastatic melanoma undergoing ICI therapy, those with sarcopenia experienced a higher incidence of grade ≥ 3 AEs compared to patients without sarcopenia, with anemia and fatigue being the most common AEs [28]. A meta-analysis revealed that there was no significant increase in the incidence of immune-related AEs (irAEs) in patients with sarcopenia after receiving immunotherapy [29]. In patients with unresectable metastatic UC undergoing ICI therapy, there was no significant correlation between sarcopenia status and the incidence of irAEs (univariate analysis, *p* = 0.1) [30]. The researchers postulated that patients without sarcopenia might exhibit higher immune activity, thereby rendering them more susceptible to the occurrence of irAEs. There is also no consensus on tumor response. Among advanced UC patients treated with ICI, the ORR was significantly higher in those without sarcopenia compared to those with sarcopenia (*p* = 0.019) [31]. However, according to Klatte et al., there was no association between pre-NAC sarcopenia and tumor response (*p* = 0.65) in muscle-invasive bladder cancer patients [32].

To our knowledge, this study is the first to focus on the relationship between pre-existing sarcopenia in UC patients receiving T + GC and hematological toxicity as well as tumor response. In this study, a total of 112 patients with UC underwent T + GC therapy for at least two cycles. Patients with sarcopenia were older, more likely to have hypertension, and had poorer ECOG-PS. In addition, we observed that patients with sarcopenia were more prone to experiencing leukopenia following T + GC therapy. At present, we have not been able to fully ascertain the potential reasons for this correlation and additional research is needed to explain this discrepancy. In terms of tumor response, we did not observe a statistically significant difference between the group of patients with sarcopenia and the group without sarcopenia. This may be due to the small sample size. Assessing patients' body composition is of significant relevance in guiding clinicians to select those who are most likely to achieve minimal hematological toxicity when considering T + GC therapy. For patients with sarcopenia, clinicians should monitor blood routine, particularly WBC count, more frequently to promptly adjust medication dosages and avoid treatment-related severe toxicities. Grade 3–4 hematological toxicity can be prevented by regular monitoring or therapeutic interventions. Whether patients with pre-existing sarcopenia can potentially prevent the occurrence of high-grade hematological toxicity through appropriate dietary interventions and exercise is a focus of our future research.

Our study has some limitations. Firstly, due to the small sample size of the retrospective study from a single institution, especially in the subgroup analysis, selection bias is inevitable. Secondly, due to the multitude of measurement methods and indices for assessing sarcopenia, making it challenging to select an appropriate cut-off value, prospective multicenter studies are required to establish clear standards and tools in this regard. Thirdly, no clear data are available on the optimal schedule for administering tislelizumab and GC chemotherapy. Finally, tumor response rate does not always indicate the potential survival benefits for patients. However, due to limitations in sample size and follow-up duration, we are currently unable to accurately assess the impact of sarcopenia on PFS and OS in patients with UC.

## Conclusion

Patients with sarcopenia were more likely to develop leukopenia after receiving at least two cycles of T + GC. No significant difference was found between the sarcopenia group and non-sarcopenia group in terms of tumor response and grade 3–4 hematological toxicity. For patients with sarcopenia who are prone to myelosuppression, we should fully inform them of the risks before the start of T + GC and be vigilant in monitoring blood tests closely.

## Data Availability

The dataset analyzed in this study is available from the corresponding author upon reasonable request.
